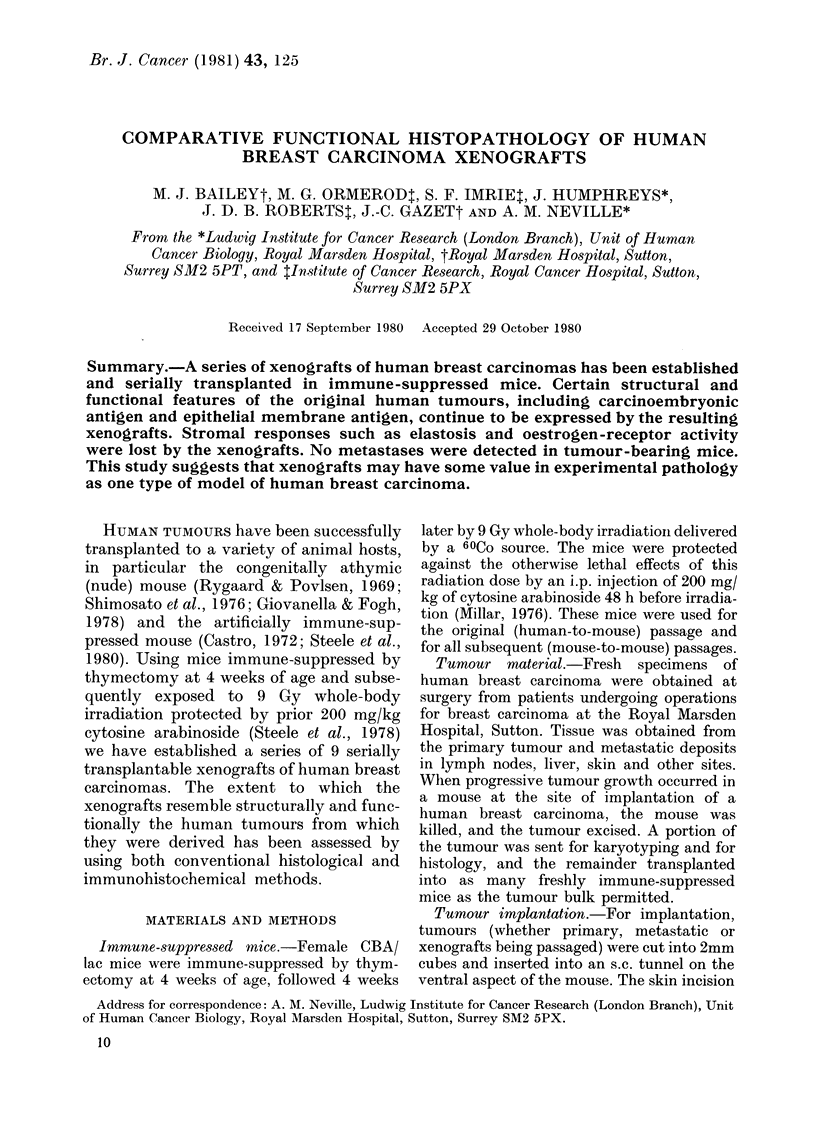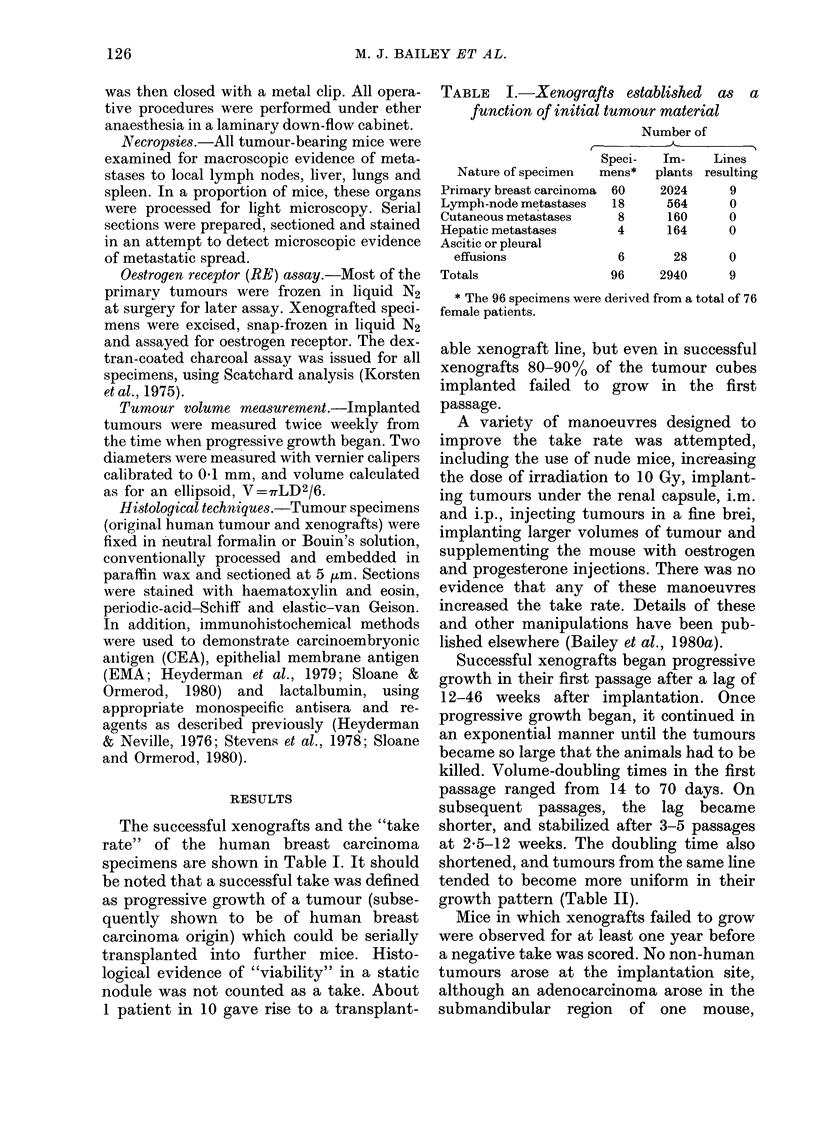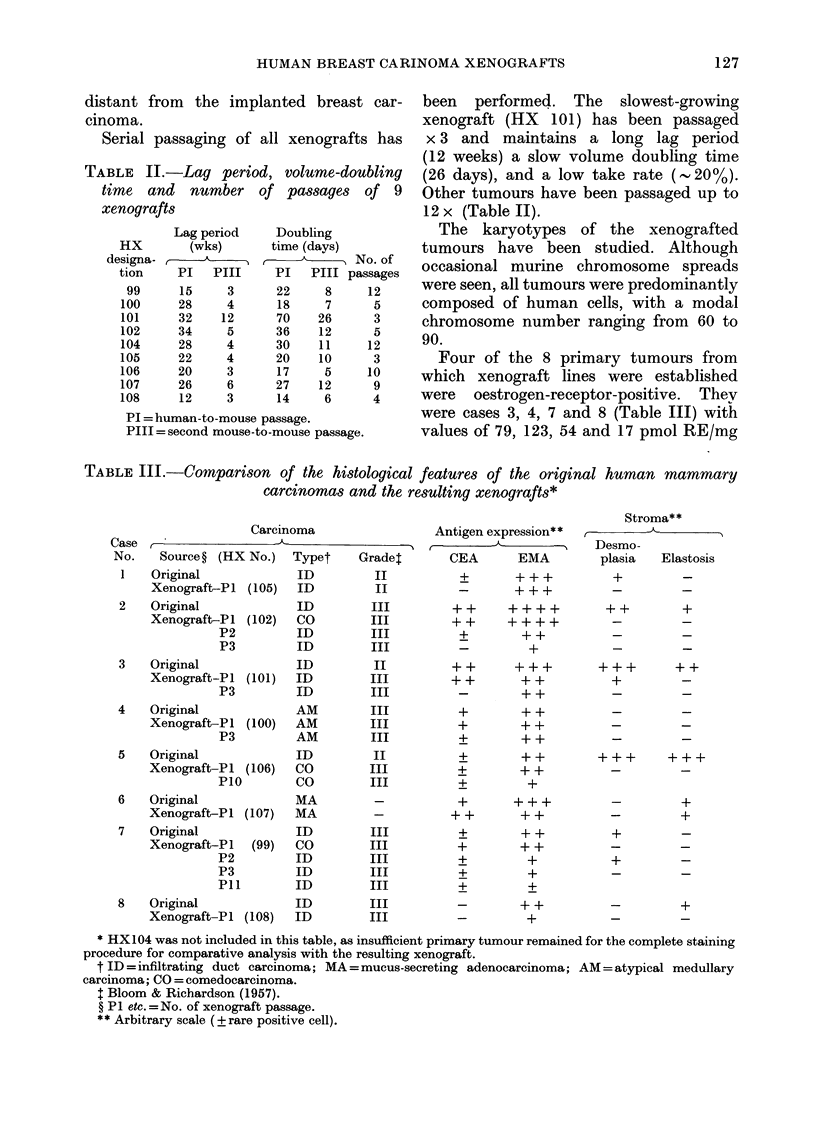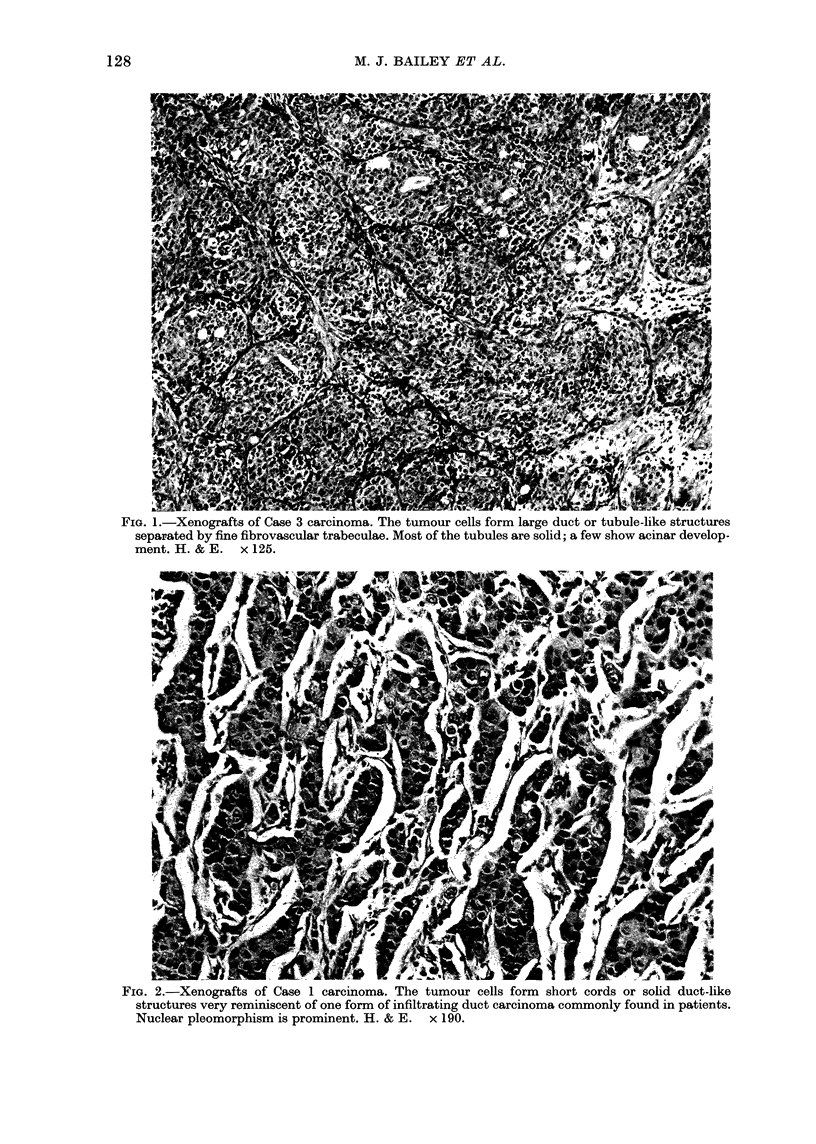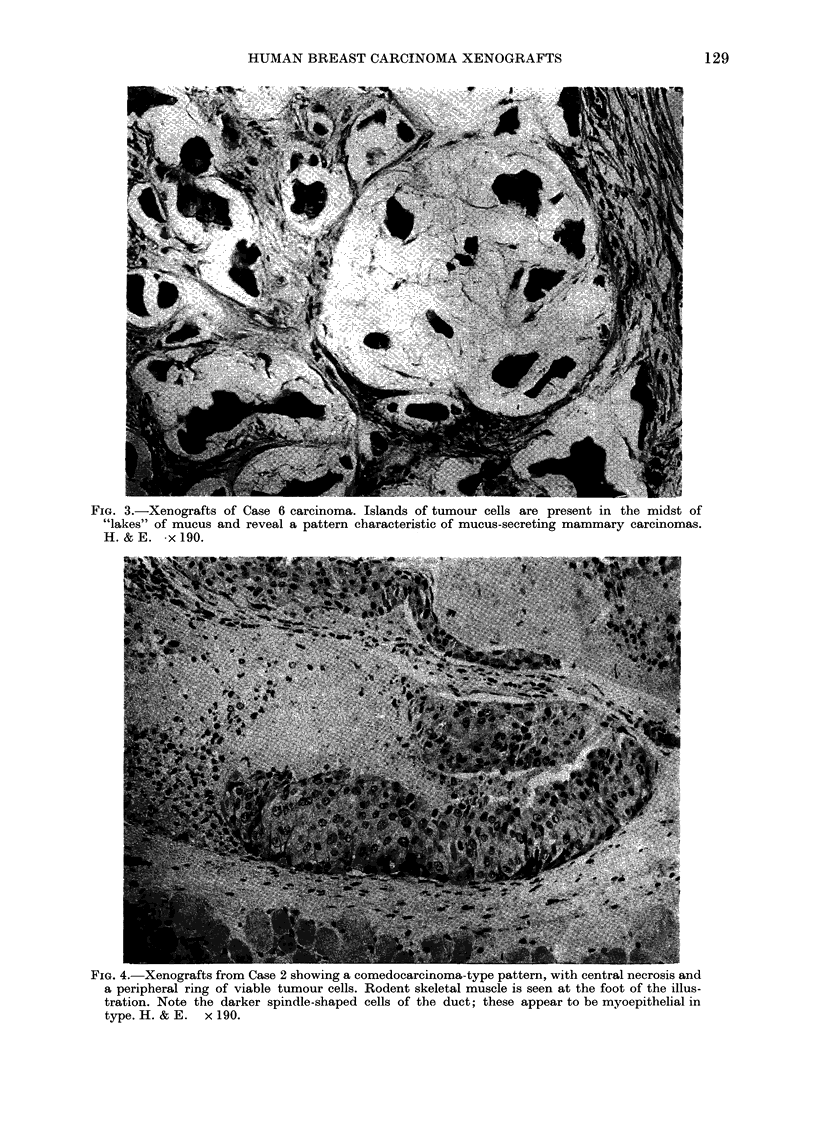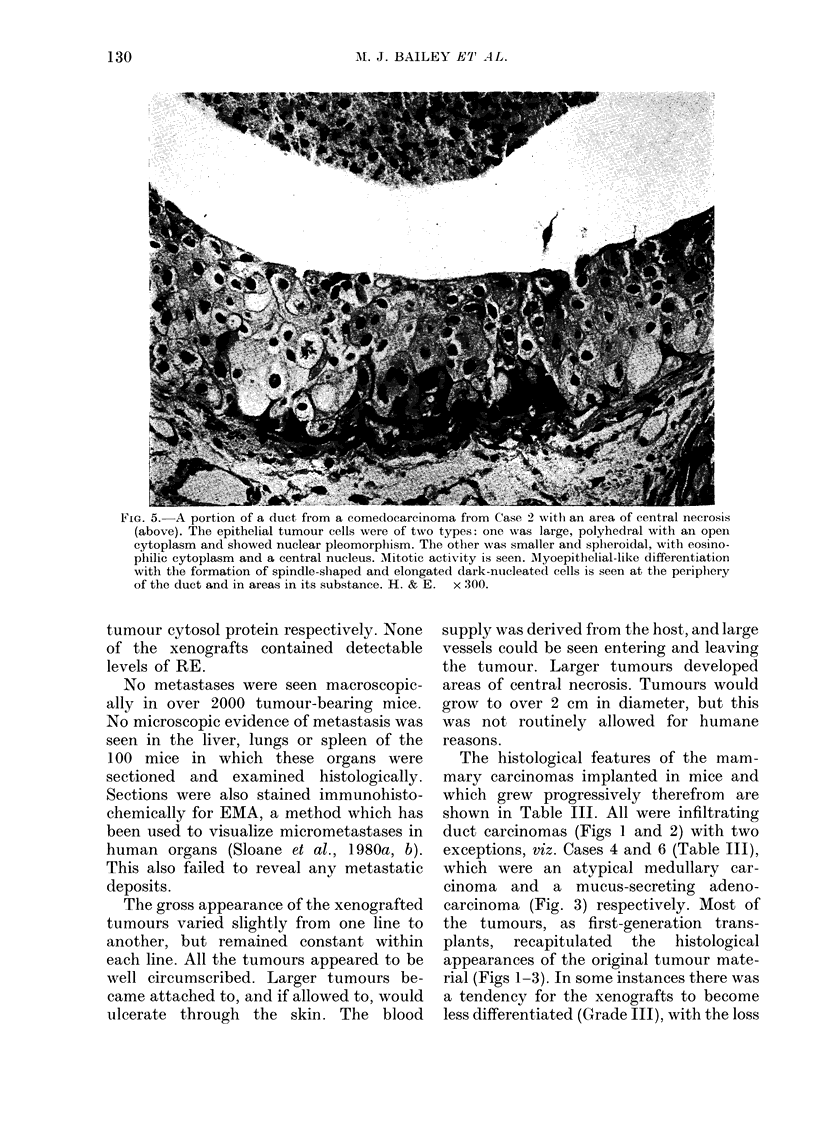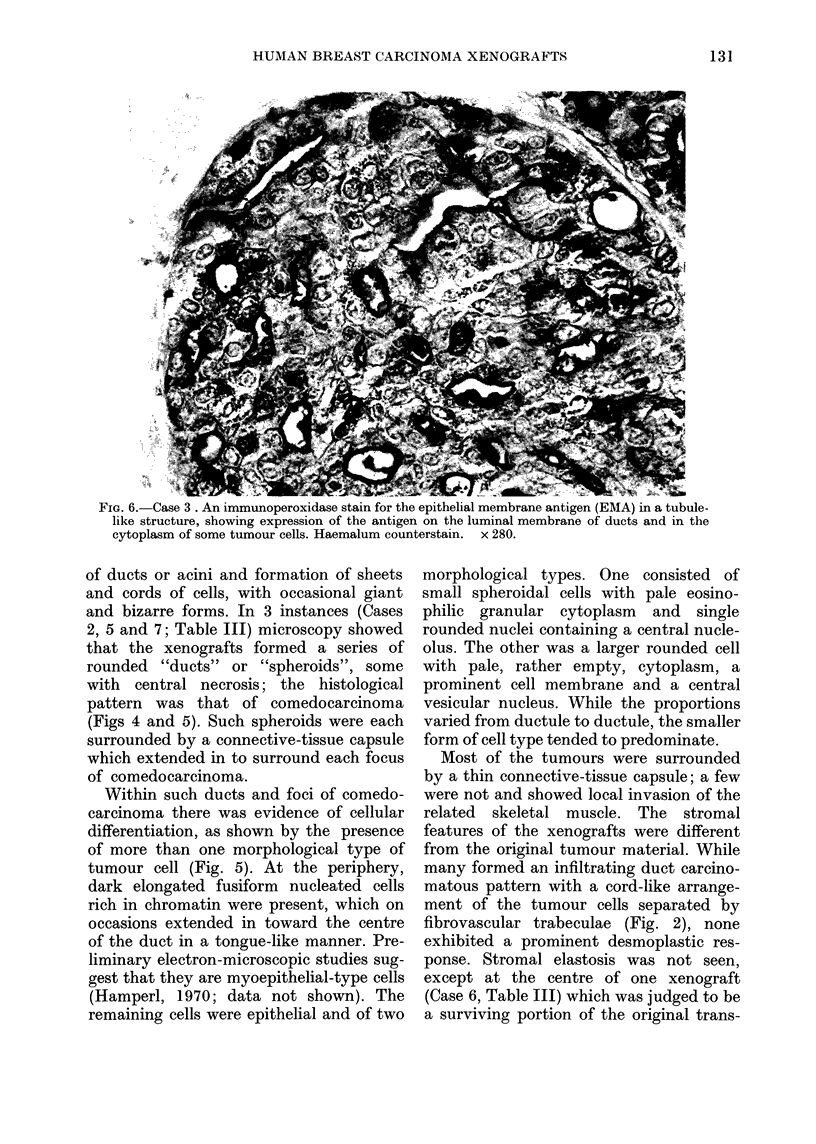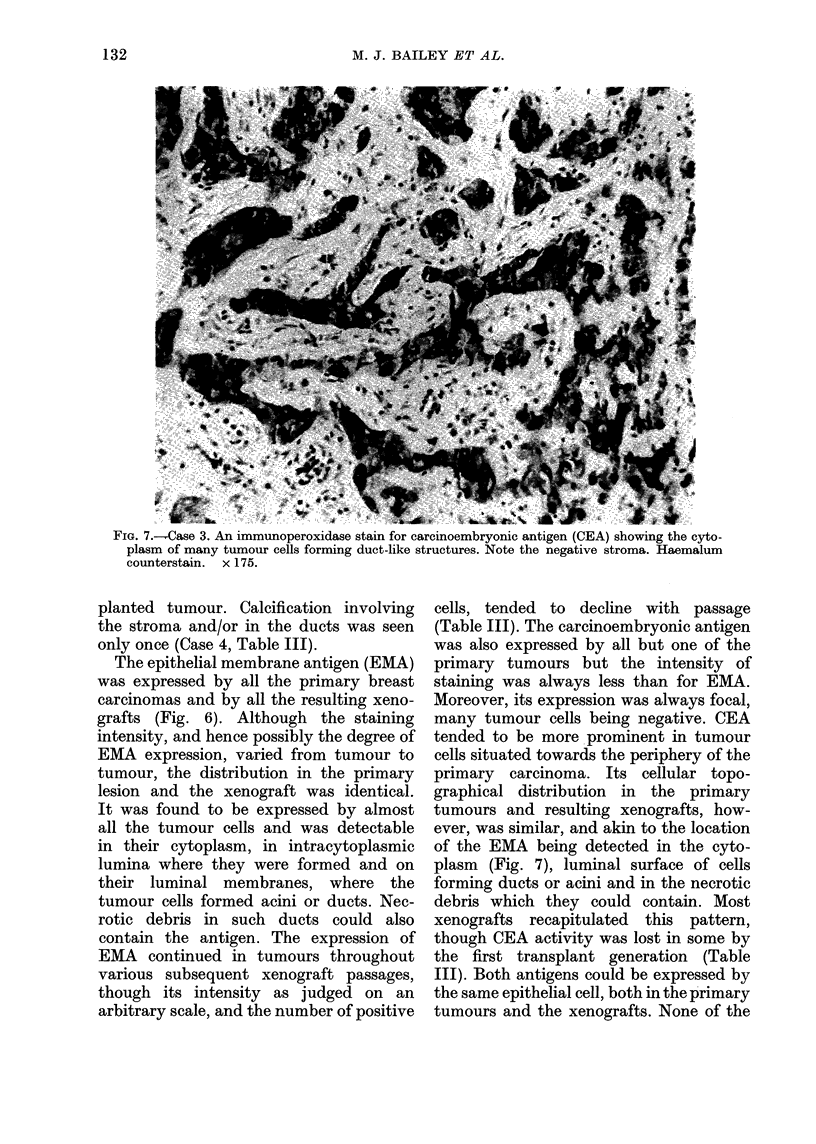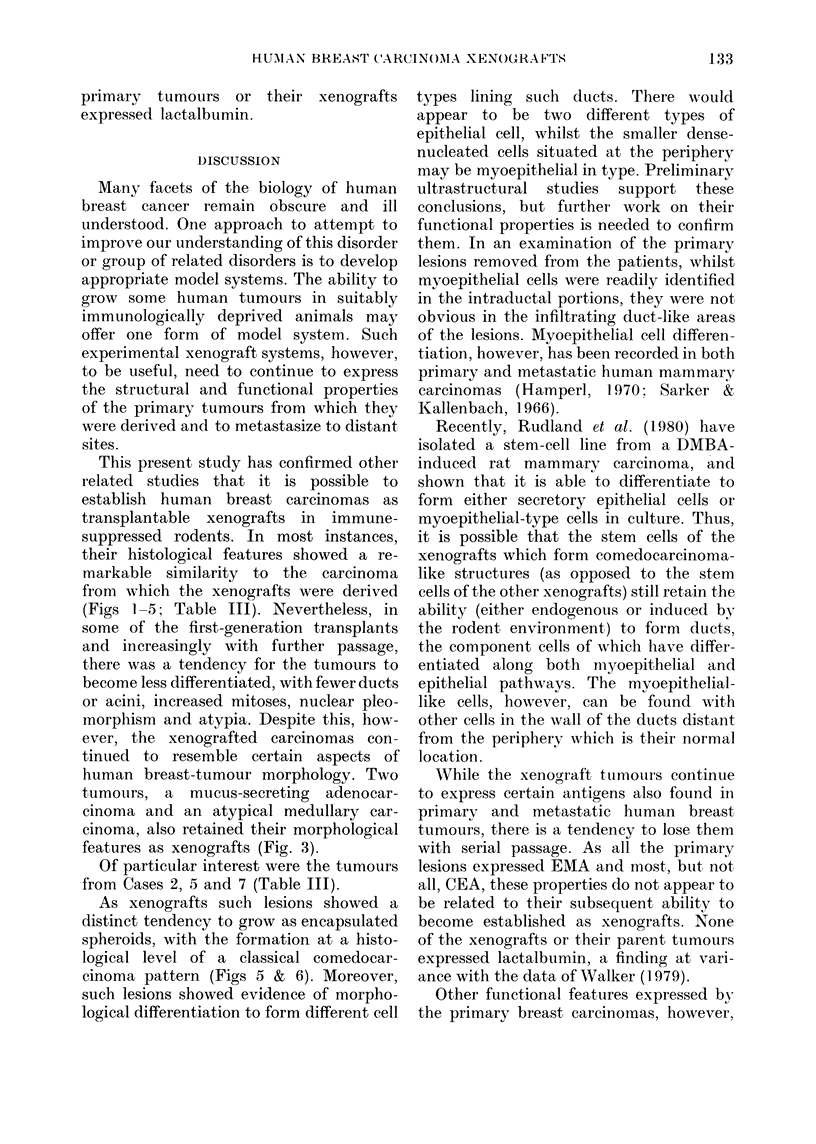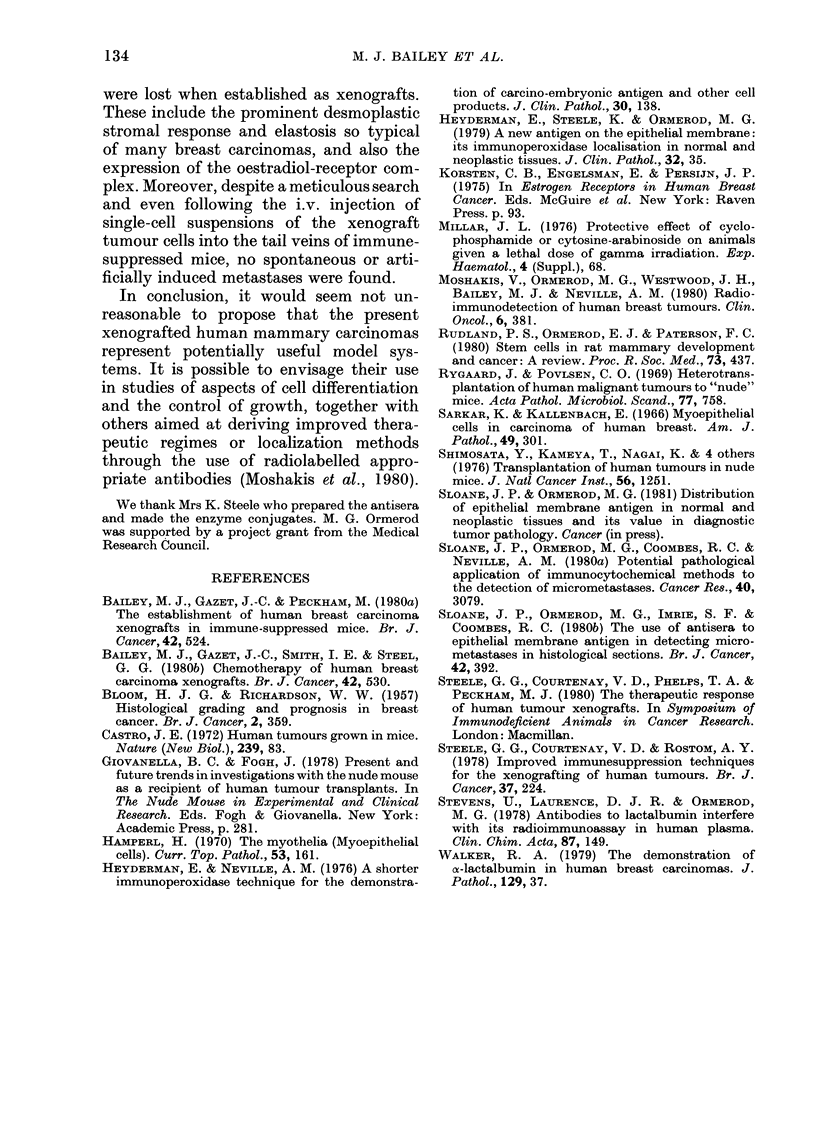# Comparative functional histopathology of human breast carcinoma xenografts.

**DOI:** 10.1038/bjc.1981.20

**Published:** 1981-02

**Authors:** M. J. Bailey, M. G. Ormerod, S. F. Imrie, J. Humphreys, J. D. Roberts, J. C. Gazet, A. M. Neville

## Abstract

**Images:**


*Br. J. Cancer* (1981) **43**, 125

### COMPARATIVE FUNCTIONAL HISTOPATHOLOGY OF HUMAN BREAST CARCINOMA XENOGRAFTS

M. J. BAILEY†, M. G. ORMEROD‡, S. F. IMRIE‡, J. HUMPHREYS*, J. D. B. ROBERTS‡, J.-C. GAZET† AND A. M. NEVILLE*

*From the *Ludwig Institute for Cancer Research (London Branch), Unit of Human Cancer Biology, Royal Marsden Hospital, †Royal Marsden Hospital, Sutton, Surrey SM2 5PT, and ‡Institute of Cancer Research, Royal Cancer Hospital, Sutton, Surrey SM2 5PX*

Received 17 September 1980 Accepted 29 October 1980

**Summary.—A series of xenografts of human breast carcinomas has been established and serially transplanted in immune-suppressed mice. Certain structural and functional features of the original human tumours, including carcinoembryonic antigen and epithelial membrane antigen, continue to be expressed by the resulting xenografts. Stromal responses such as elastosis and oestrogen-receptor activity were lost by the xenografts. No metastases were detected in tumour-bearing mice. This study suggests that xenografts may have some value in experimental pathology as one type of model of human breast carcinoma.**

HUMAN TUMOURS have been successfully transplanted to a variety of animal hosts, in particular the congenitally athymic (nude) mouse (Rygaard & Povlsen, 1969; Shimosato *et al.*, 1976; Giovanella & Fogh, 1978) and the artificially immune-suppressed mouse (Castro, 1972; Steele *et al.*, 1980). Using mice immune-suppressed by thymectomy at 4 weeks of age and subsequently exposed to 9 Gy whole-body irradiation protected by prior 200 mg/kg cytosine arabinoside (Steele *et al.*, 1978) we have established a series of 9 serially transplantable xenografts of human breast carcinomas. The extent to which the xenografts resemble structurally and functionally the human tumours from which they were derived has been assessed by using both conventional histological and immunohistochemical methods.

### MATERIALS AND METHODS

*Immune-suppressed mice.*—Female CBA/lac mice were immune-suppressed by thymectomy at 4 weeks of age, followed 4 weeks later by 9 Gy whole-body irradiation delivered by a $^{60}$Co source. The mice were protected against the otherwise lethal effects of this radiation dose by an i.p. injection of 200 mg/kg of cytosine arabinoside 48 h before irradiation (Millar, 1976). These mice were used for the original (human-to-mouse) passage and for all subsequent (mouse-to-mouse) passages.

*Tumour material.*—Fresh specimens of human breast carcinoma were obtained at surgery from patients undergoing operations for breast carcinoma at the Royal Marsden Hospital, Sutton. Tissue was obtained from the primary tumour and metastatic deposits in lymph nodes, liver, skin and other sites. When progressive tumour growth occurred in a mouse at the site of implantation of a human breast carcinoma, the mouse was killed, and the tumour excised. A portion of the tumour was sent for karyotyping and for histology, and the remainder transplanted into as many freshly immune-suppressed mice as the tumour bulk permitted.

*Tumour implantation.*—For implantation, tumours (whether primary, metastatic or xenografts being passaged) were cut into 2mm cubes and inserted into an s.c. tunnel on the ventral aspect of the mouse. The skin incision

Address for correspondence: A. M. Neville, Ludwig Institute for Cancer Research (London Branch), Unit of Human Cancer Biology, Royal Marsden Hospital, Sutton, Surrey SM2 5PX.

was then closed with a metal clip. All operative procedures were performed under ether anaesthesia in a laminary down-flow cabinet.

*Necropsies.*—All tumour-bearing mice were examined for macroscopic evidence of metastases to local lymph nodes, liver, lungs and spleen. In a proportion of mice, these organs were processed for light microscopy. Serial sections were prepared, sectioned and stained in an attempt to detect microscopic evidence of metastatic spread.

*Oestrogen receptor (RE) assay.*—Most of the primary tumours were frozen in liquid $N_2$ at surgery for later assay. Xenografted specimens were excised, snap-frozen in liquid $N_2$ and assayed for oestrogen receptor. The dextran-coated charcoal assay was issued for all specimens, using Scatchard analysis (Korsten *et al.*, 1975).

*Tumour volume measurement.*—Implanted tumours were measured twice weekly from the time when progressive growth began. Two diameters were measured with vernier calipers calibrated to 0·1 mm, and volume calculated as for an ellipsoid, $V = \pi LD^2/6$.

*Histological techniques.*—Tumour specimens (original human tumour and xenografts) were fixed in neutral formalin or Bouin's solution, conventionally processed and embedded in paraffin wax and sectioned at 5 μm. Sections were stained with haematoxylin and eosin, periodic-acid–Schiff and elastic–van Geison. In addition, immunohistochemical methods were used to demonstrate carcinoembryonic antigen (CEA), epithelial membrane antigen (EMA; Heyderman *et al.*, 1979; Sloane & Ormerod, 1980) and lactalbumin, using appropriate monospecific antisera and reagents as described previously (Heyderman & Neville, 1976; Stevens *et al.*, 1978; Sloane and Ormerod, 1980).

### RESULTS

The successful xenografts and the "take rate" of the human breast carcinoma specimens are shown in Table I. It should be noted that a successful take was defined as progressive growth of a tumour (subsequently shown to be of human breast carcinoma origin) which could be serially transplanted into further mice. Histological evidence of "viability" in a static nodule was not counted as a take. About 1 patient in 10 gave rise to a transplantable xenograft line, but even in successful xenografts 80–90% of the tumour cubes implanted failed to grow in the first passage.

TABLE I.—*Xenografts established as a function of initial tumour material*

| | Number of | | |
|---|---|---|---|
| Nature of specimen | Specimens* | Implants | Lines resulting |
| Primary breast carcinoma | 60 | 2024 | 9 |
| Lymph-node metastases | 18 | 564 | 0 |
| Cutaneous metastases | 8 | 160 | 0 |
| Hepatic metastases | 4 | 164 | 0 |
| Ascitic or pleural effusions | 6 | 28 | 0 |
| Totals | 96 | 2940 | 9 |

* The 96 specimens were derived from a total of 76 female patients.

A variety of manoeuvres designed to improve the take rate was attempted, including the use of nude mice, increasing the dose of irradiation to 10 Gy, implanting tumours under the renal capsule, i.m. and i.p., injecting tumours in a fine brei, implanting larger volumes of tumour and supplementing the mouse with oestrogen and progesterone injections. There was no evidence that any of these manoeuvres increased the take rate. Details of these and other manipulations have been published elsewhere (Bailey *et al.*, 1980*a*).

Successful xenografts began progressive growth in their first passage after a lag of 12–46 weeks after implantation. Once progressive growth began, it continued in an exponential manner until the tumours became so large that the animals had to be killed. Volume-doubling times in the first passage ranged from 14 to 70 days. On subsequent passages, the lag became shorter, and stabilized after 3–5 passages at 2·5–12 weeks. The doubling time also shortened, and tumours from the same line tended to become more uniform in their growth pattern (Table II).

Mice in which xenografts failed to grow were observed for at least one year before a negative take was scored. No non-human tumours arose at the implantation site, although an adenocarcinoma arose in the submandibular region of one mouse, distant from the implanted breast carcinoma.

Serial passaging of all xenografts has been performed. The slowest-growing xenograft (HX 101) has been passaged ×3 and maintains a long lag period (12 weeks) a slow volume doubling time (26 days), and a low take rate (~20%). Other tumours have been passaged up to 12× (Table II).

TABLE II.—*Lag period, volume-doubling time and number of passages of 9 xenografts*

| HX designation | Lag period (wks) PI | Lag period (wks) PIII | Doubling time (days) PI | Doubling time (days) PIII | No. of passages |
|---|---|---|---|---|---|
| 99 | 15 | 3 | 22 | 8 | 12 |
| 100 | 28 | 4 | 18 | 7 | 5 |
| 101 | 32 | 12 | 70 | 26 | 3 |
| 102 | 34 | 5 | 36 | 12 | 5 |
| 104 | 28 | 4 | 30 | 11 | 12 |
| 105 | 22 | 4 | 20 | 10 | 3 |
| 106 | 20 | 3 | 17 | 5 | 10 |
| 107 | 26 | 6 | 27 | 12 | 9 |
| 108 | 12 | 3 | 14 | 6 | 4 |

PI = human-to-mouse passage.
PIII = second mouse-to-mouse passage.

The karyotypes of the xenografted tumours have been studied. Although occasional murine chromosome spreads were seen, all tumours were predominantly composed of human cells, with a modal chromosome number ranging from 60 to 90.

Four of the 8 primary tumours from which xenograft lines were established were oestrogen-receptor-positive. They were cases 3, 4, 7 and 8 (Table III) with values of 79, 123, 54 and 17 pmol RE/mg

TABLE III.—*Comparison of the histological features of the original human mammary carcinomas and the resulting xenografts**

| Case No. | Carcinoma Source§ | (HX No.) | Type† | Grade‡ | Antigen expression** CEA | EMA | Stroma** Desmoplasia | Elastosis |
|---|---|---|---|---|---|---|---|---|
| 1 | Original | | ID | II | ± | +++ | + | − |
| | Xenograft–P1 | (105) | ID | II | − | +++ | − | − |
| 2 | Original | | ID | III | ++ | ++++ | ++ | + |
| | Xenograft–P1 | (102) | CO | III | ++ | ++++ | − | − |
| | P2 | | ID | III | ± | ++ | − | − |
| | P3 | | ID | III | − | + | − | − |
| 3 | Original | | ID | II | ++ | +++ | +++ | ++ |
| | Xenograft–P1 | (101) | ID | III | ++ | ++ | + | − |
| | P3 | | ID | III | − | ++ | − | − |
| 4 | Original | | AM | III | + | ++ | − | − |
| | Xenograft–P1 | (100) | AM | III | + | ++ | − | − |
| | P3 | | AM | III | ± | ++ | − | − |
| 5 | Original | | ID | II | ± | ++ | +++ | +++ |
| | Xenograft–P1 | (106) | CO | III | ± | ++ | − | − |
| | P10 | | CO | III | ± | + | | |
| 6 | Original | | MA | − | + | +++ | − | + |
| | Xenograft–P1 | (107) | MA | − | ++ | ++ | − | + |
| 7 | Original | | ID | III | ± | ++ | + | − |
| | Xenograft–P1 | (99) | CO | III | + | ++ | − | − |
| | P2 | | ID | III | ± | + | + | − |
| | P3 | | ID | III | ± | + | − | − |
| | P11 | | ID | III | ± | ± | | |
| 8 | Original | | ID | III | − | ++ | − | + |
| | Xenograft–P1 | (108) | ID | III | − | + | − | − |

* HX104 was not included in this table, as insufficient primary tumour remained for the complete staining procedure for comparative analysis with the resulting xenograft.

† ID = infiltrating duct carcinoma; MA = mucus-secreting adenocarcinoma; AM = atypical medullary carcinoma; CO = comedocarcinoma.

‡ Bloom & Richardson (1957).

§ P1 *etc.* = No. of xenograft passage.

** Arbitrary scale (± rare positive cell).

FIG. 1.—Xenografts of Case 3 carcinoma. The tumour cells form large duct or tubule-like structures separated by fine fibrovascular trabeculae. Most of the tubules are solid; a few show acinar development. H. & E. ×125.

FIG. 2.—Xenografts of Case 1 carcinoma. The tumour cells form short cords or solid duct-like structures very reminiscent of one form of infiltrating duct carcinoma commonly found in patients. Nuclear pleomorphism is prominent. H. & E. ×190.

FIG. 3.—Xenografts of Case 6 carcinoma. Islands of tumour cells are present in the midst of "lakes" of mucus and reveal a pattern characteristic of mucus-secreting mammary carcinomas. H. & E. ×190.

FIG. 4.—Xenografts from Case 2 showing a comedocarcinoma-type pattern, with central necrosis and a peripheral ring of viable tumour cells. Rodent skeletal muscle is seen at the foot of the illustration. Note the darker spindle-shaped cells of the duct; these appear to be myoepithelial in type. H. & E. ×190.

FIG. 5.—A portion of a duct from a comedocarcinoma from Case 2 with an area of central necrosis (above). The epithelial tumour cells were of two types: one was large, polyhedral with an open cytoplasm and showed nuclear pleomorphism. The other was smaller and spheroidal, with eosinophilic cytoplasm and a central nucleus. Mitotic activity is seen. Myoepithelial-like differentiation with the formation of spindle-shaped and elongated dark-nucleated cells is seen at the periphery of the duct and in areas in its substance. H. & E. ×300.

tumour cytosol protein respectively. None of the xenografts contained detectable levels of RE.

No metastases were seen macroscopically in over 2000 tumour-bearing mice. No microscopic evidence of metastasis was seen in the liver, lungs or spleen of the 100 mice in which these organs were sectioned and examined histologically. Sections were also stained immunohistochemically for EMA, a method which has been used to visualize micrometastases in human organs (Sloane *et al.*, 1980*a*, *b*). This also failed to reveal any metastatic deposits.

The gross appearance of the xenografted tumours varied slightly from one line to another, but remained constant within each line. All the tumours appeared to be well circumscribed. Larger tumours became attached to, and if allowed to, would ulcerate through the skin. The blood supply was derived from the host, and large vessels could be seen entering and leaving the tumour. Larger tumours developed areas of central necrosis. Tumours would grow to over 2 cm in diameter, but this was not routinely allowed for humane reasons.

The histological features of the mammary carcinomas implanted in mice and which grew progressively therefrom are shown in Table III. All were infiltrating duct carcinomas (Figs 1 and 2) with two exceptions, *viz.* Cases 4 and 6 (Table III), which were an atypical medullary carcinoma and a mucus-secreting adenocarcinoma (Fig. 3) respectively. Most of the tumours, as first-generation transplants, recapitulated the histological appearances of the original tumour material (Figs 1–3). In some instances there was a tendency for the xenografts to become less differentiated (Grade III), with the loss

FIG. 6.—Case 3 . An immunoperoxidase stain for the epithelial membrane antigen (EMA) in a tubule-like structure, showing expression of the antigen on the luminal membrane of ducts and in the cytoplasm of some tumour cells. Haemalum counterstain. × 280.

of ducts or acini and formation of sheets and cords of cells, with occasional giant and bizarre forms. In 3 instances (Cases 2, 5 and 7; Table III) microscopy showed that the xenografts formed a series of rounded "ducts" or "spheroids", some with central necrosis; the histological pattern was that of comedocarcinoma (Figs 4 and 5). Such spheroids were each surrounded by a connective-tissue capsule which extended in to surround each focus of comedocarcinoma.

Within such ducts and foci of comedocarcinoma there was evidence of cellular differentiation, as shown by the presence of more than one morphological type of tumour cell (Fig. 5). At the periphery, dark elongated fusiform nucleated cells rich in chromatin were present, which on occasions extended in toward the centre of the duct in a tongue-like manner. Preliminary electron-microscopic studies suggest that they are myoepithelial-type cells (Hamperl, 1970; data not shown). The remaining cells were epithelial and of two morphological types. One consisted of small spheroidal cells with pale eosinophilic granular cytoplasm and single rounded nuclei containing a central nucleolus. The other was a larger rounded cell with pale, rather empty, cytoplasm, a prominent cell membrane and a central vesicular nucleus. While the proportions varied from ductule to ductule, the smaller form of cell type tended to predominate.

Most of the tumours were surrounded by a thin connective-tissue capsule; a few were not and showed local invasion of the related skeletal muscle. The stromal features of the xenografts were different from the original tumour material. While many formed an infiltrating duct carcinomatous pattern with a cord-like arrangement of the tumour cells separated by fibrovascular trabeculae (Fig. 2), none exhibited a prominent desmoplastic response. Stromal elastosis was not seen, except at the centre of one xenograft (Case 6, Table III) which was judged to be a surviving portion of the original trans-

FIG. 7.—Case 3. An immunoperoxidase stain for carcinoembryonic antigen (CEA) showing the cytoplasm of many tumour cells forming duct-like structures. Note the negative stroma. Haemalum counterstain. ×175.

planted tumour. Calcification involving the stroma and/or in the ducts was seen only once (Case 4, Table III).

The epithelial membrane antigen (EMA) was expressed by all the primary breast carcinomas and by all the resulting xenografts (Fig. 6). Although the staining intensity, and hence possibly the degree of EMA expression, varied from tumour to tumour, the distribution in the primary lesion and the xenograft was identical. It was found to be expressed by almost all the tumour cells and was detectable in their cytoplasm, in intracytoplasmic lumina where they were formed and on their luminal membranes, where the tumour cells formed acini or ducts. Necrotic debris in such ducts could also contain the antigen. The expression of EMA continued in tumours throughout various subsequent xenograft passages, though its intensity as judged on an arbitrary scale, and the number of positive cells, tended to decline with passage (Table III). The carcinoembryonic antigen was also expressed by all but one of the primary tumours but the intensity of staining was always less than for EMA. Moreover, its expression was always focal, many tumour cells being negative. CEA tended to be more prominent in tumour cells situated towards the periphery of the primary carcinoma. Its cellular topographical distribution in the primary tumours and resulting xenografts, however, was similar, and akin to the location of the EMA being detected in the cytoplasm (Fig. 7), luminal surface of cells forming ducts or acini and in the necrotic debris which they could contain. Most xenografts recapitulated this pattern, though CEA activity was lost in some by the first transplant generation (Table III). Both antigens could be expressed by the same epithelial cell, both in the primary tumours and the xenografts. None of the primary tumours or their xenografts expressed lactalbumin.

### DISCUSSION

Many facets of the biology of human breast cancer remain obscure and ill understood. One approach to attempt to improve our understanding of this disorder or group of related disorders is to develop appropriate model systems. The ability to grow some human tumours in suitably immunologically deprived animals may offer one form of model system. Such experimental xenograft systems, however, to be useful, need to continue to express the structural and functional properties of the primary tumours from which they were derived and to metastasize to distant sites.

This present study has confirmed other related studies that it is possible to establish human breast carcinomas as transplantable xenografts in immune-suppressed rodents. In most instances, their histological features showed a remarkable similarity to the carcinoma from which the xenografts were derived (Figs 1–5; Table III). Nevertheless, in some of the first-generation transplants and increasingly with further passage, there was a tendency for the tumours to become less differentiated, with fewer ducts or acini, increased mitoses, nuclear pleomorphism and atypia. Despite this, however, the xenografted carcinomas continued to resemble certain aspects of human breast-tumour morphology. Two tumours, a mucus-secreting adenocarcinoma and an atypical medullary carcinoma, also retained their morphological features as xenografts (Fig. 3).

Of particular interest were the tumours from Cases 2, 5 and 7 (Table III).

As xenografts such lesions showed a distinct tendency to grow as encapsulated spheroids, with the formation at a histological level of a classical comedocarcinoma pattern (Figs 5 & 6). Moreover, such lesions showed evidence of morphological differentiation to form different cell types lining such ducts. There would appear to be two different types of epithelial cell, whilst the smaller dense-nucleated cells situated at the periphery may be myoepithelial in type. Preliminary ultrastructural studies support these conclusions, but further work on their functional properties is needed to confirm them. In an examination of the primary lesions removed from the patients, whilst myoepithelial cells were readily identified in the intraductal portions, they were not obvious in the infiltrating duct-like areas of the lesions. Myoepithelial cell differentiation, however, has been recorded in both primary and metastatic human mammary carcinomas (Hamperl, 1970; Sarker & Kallenbach, 1966).

Recently, Rudland *et al.* (1980) have isolated a stem-cell line from a DMBA-induced rat mammary carcinoma, and shown that it is able to differentiate to form either secretory epithelial cells or myoepithelial-type cells in culture. Thus, it is possible that the stem cells of the xenografts which form comedocarcinoma-like structures (as opposed to the stem cells of the other xenografts) still retain the ability (either endogenous or induced by the rodent environment) to form ducts, the component cells of which have differentiated along both myoepithelial and epithelial pathways. The myoepithelial-like cells, however, can be found with other cells in the wall of the ducts distant from the periphery which is their normal location.

While the xenograft tumours continue to express certain antigens also found in primary and metastatic human breast tumours, there is a tendency to lose them with serial passage. As all the primary lesions expressed EMA and most, but not all, CEA, these properties do not appear to be related to their subsequent ability to become established as xenografts. None of the xenografts or their parent tumours expressed lactalbumin, a finding at variance with the data of Walker (1979).

Other functional features expressed by the primary breast carcinomas, however, were lost when established as xenografts. These include the prominent desmoplastic stromal response and elastosis so typical of many breast carcinomas, and also the expression of the oestradiol-receptor complex. Moreover, despite a meticulous search and even following the i.v. injection of single-cell suspensions of the xenograft tumour cells into the tail veins of immune-suppressed mice, no spontaneous or artificially induced metastases were found.

In conclusion, it would seem not unreasonable to propose that the present xenografted human mammary carcinomas represent potentially useful model systems. It is possible to envisage their use in studies of aspects of cell differentiation and the control of growth, together with others aimed at deriving improved therapeutic regimes or localization methods through the use of radiolabelled appropriate antibodies (Moshakis *et al.*, 1980).

We thank Mrs K. Steele who prepared the antisera and made the enzyme conjugates. M. G. Ormerod was supported by a project grant from the Medical Research Council.

REFERENCES

BAILEY, M. J., GAZET, J.-C. & PECKHAM, M. (1980*a*) The establishment of human breast carcinoma xenografts in immune-suppressed mice. *Br. J. Cancer*, **42**, 524.

BAILEY, M. J., GAZET, J.-C., SMITH, I. E. & STEEL, G. G. (1980*b*) Chemotherapy of human breast carcinoma xenografts. *Br. J. Cancer*, **42**, 530.

BLOOM, H. J. G. & RICHARDSON, W. W. (1957) Histological grading and prognosis in breast cancer. *Br. J. Cancer*, **2**, 359.

CASTRO, J. E. (1972) Human tumours grown in mice. *Nature* (*New Biol.*), **239**, 83.

GIOVANELLA, B. C. & FOGH, J. (1978) Present and future trends in investigations with the nude mouse as a recipient of human tumour transplants. In *The Nude Mouse in Experimental and Clinical Research*. Eds. Fogh & Giovanella. New York: Academic Press, p. 281.

HAMPERL, H. (1970) The myothelia (Myoepithelial cells). *Curr. Top. Pathol.*, **53**, 161.

HEYDERMAN, E. & NEVILLE, A. M. (1976) A shorter immunoperoxidase technique for the demonstration of carcino-embryonic antigen and other cell products. *J. Clin. Pathol.*, **30**, 138.

HEYDERMAN, E., STEELE, K. & ORMEROD, M. G. (1979) A new antigen on the epithelial membrane: its immunoperoxidase localisation in normal and neoplastic tissues. *J. Clin. Pathol.*, **32**, 35.

KORSTEN, C. B., ENGELSMAN, E. & PERSIJN, J. P. (1975) In *Estrogen Receptors in Human Breast Cancer*. Eds. McGuire *et al.* New York: Raven Press. p. 93.

MILLAR, J. L. (1976) Protective effect of cyclophosphamide or cytosine-arabinoside on animals given a lethal dose of gamma irradiation. *Exp. Haematol.*, **4** (Suppl.), 68.

MOSHAKIS, V., ORMEROD, M. G., WESTWOOD, J. H., BAILEY, M. J. & NEVILLE, A. M. (1980) Radioimmunodetection of human breast tumours. *Clin. Oncol.*, **6**, 381.

RUDLAND, P. S., ORMEROD, E. J. & PATERSON, F. C. (1980) Stem cells in rat mammary development and cancer: A review. *Proc. R. Soc. Med.*, **73**, 437.

RYGAARD, J. & POVLSEN, C. O. (1969) Heterotransplantation of human malignant tumours to "nude" mice. *Acta Pathol. Microbiol. Scand.*, **77**, 758.

SARKAR, K. & KALLENBACH, E. (1966) Myoepithelial cells in carcinoma of human breast. *Am. J. Pathol.*, **49**, 301.

SHIMOSATA, Y., KAMEYA, T., NAGAI, K. & 4 others (1976) Transplantation of human tumours in nude mice. *J. Natl Cancer Inst.*, **56**, 1251.

SLOANE, J. P. & ORMEROD, M. G. (1981) Distribution of epithelial membrane antigen in normal and neoplastic tissues and its value in diagnostic tumor pathology. *Cancer* (in press).

SLOANE, J. P., ORMEROD, M. G., COOMBES, R. C. & NEVILLE, A. M. (1980*a*) Potential pathological application of immunocytochemical methods to the detection of micrometastases. *Cancer Res.*, **40**, 3079.

SLOANE, J. P., ORMEROD, M. G., IMRIE, S. F. & COOMBES, R. C. (1980*b*) The use of antisera to epithelial membrane antigen in detecting micrometastases in histological sections. *Br. J. Cancer*, **42**, 392.

STEELE, G. G., COURTENAY, V. D., PHELPS, T. A. & PECKHAM, M. J. (1980) The therapeutic response of human tumour xenografts. In *Symposium of Immunodeficient Animals in Cancer Research*. London: Macmillan.

STEELE, G. G., COURTENAY, V. D. & ROSTOM, A. Y. (1978) Improved immunesuppression techniques for the xenografting of human tumours. *Br. J. Cancer*, **37**, 224.

STEVENS, U., LAURENCE, D. J. R. & ORMEROD, M. G. (1978) Antibodies to lactalbumin interfere with its radioimmunoassay in human plasma. *Clin. Chim. Acta*, **87**, 149.

WALKER, R. A. (1979) The demonstration of α-lactalbumin in human breast carcinomas. *J. Pathol.*, **129**, 37.